# Draft Genome Sequence of *Cyanobacterium* sp. Strain IPPAS B-1200 with a Unique Fatty Acid Composition

**DOI:** 10.1128/genomeA.01306-16

**Published:** 2016-11-17

**Authors:** Alexander Y. Starikov, Aizhan A. Usserbaeva, Maria A. Sinetova, Fariza K. Sarsekeyeva, Bolatkhan K. Zayadan, Vera V. Ustinova, Elena V. Kupriyanova, Dmitry A. Los, Kirill S. Mironov

**Affiliations:** a Institute of Plant Physiology, Russian Academy of Science, Moscow, Russia; b Department of Biotechnology, Faculty of Biology and Biotechnology, Al-Farabi Kazakh National University, Almaty, Kazakhstan; c Microbiology Department, Central Tuberculosis Research Institute RAMS, Moscow, Russia

## Abstract

Here, we report the draft genome of *Cyanobacterium* sp. IPPAS strain B-1200, isolated from Lake Balkhash, Kazakhstan, and characterized by the unique fatty acid composition of its membrane lipids, which are enriched with myristic and myristoleic acids. The approximate genome size is 3.4 Mb, and the predicted number of coding sequences is 3,119.

## GENOME ANNOUNCEMENT

Cyanobacteria are a prolific source of various useful compounds, including fatty acids suitable for biodiesel production (1). The cyanobacterial strain *Cyanobacterium* sp. IPPAS B-1200 has been characterized by the unique fatty acid composition of its membrane lipids, which are enriched with myristic (30%), myristoleic (10%), palmitic (40%), and palmitoleic (10%) acids (2). The strain was able to grow intensively at temperatures ranging from 24 to 39°C. Such characteristics make this strain a target candidate for production of biodiesel with a high cetane number.

Genomic DNA was isolated from cyanobacterial cells by incubation with saturated iodide solution followed by lysozyme treatment and 2% SDS lysis at 70°C (3). Lysate was treated with a phenol-chloroform mixture for DNA purification. DNA libraries for sequencing were prepared using Nextera XT and Ion Xpress kits. Sequencing was performed on the MiSeq system with MiSeq reagent kit version 3 in a 600-cycle paired-end format and on the Ion PGM system with Hi-Q chemistry.

The genome was assembled using SPAdes version 3.7.1 (4) and MIRA version 4.0.2 software (5). The draft genome assembly quality was analyzed by QUAST (6). The draft genome’s median coverage was approximately 200× and its *N*_50_ value was 80,222 bp. The approximate genome size is 3.4 Mb, with an estimated average G+C content of 37.7%.

The genome was annotated using the automated NCBI Prokaryotic Genome Annotation Pipeline (PGAP). It contained a total of 3,119 genes, with 2,934 genes coding for proteins, 137 pseudogenes, four rRNA-coding sequences, 40 tRNAs, and four noncoding RNAs. Two CRISPR arrays were found in the genome. The genome contained only one gene for the acyl-lipid Δ9-desaturase *desC*, which is responsible for Δ^9^-dehydrogenation of C14 and C16 fatty acids. This coincides with genomic data of the type strain of this genus, *Cyanobacterium stanierii* PCC 7202 (7). Therefore, *Cyanobacterium* sp. strain IPPAS B-1200 is an atypical representative of cyanobacteria of group 1 that containing only monounsaturated fatty acids in both the *sn*-1 and *sn*-2 positions of membrane lipids (8).

### Accession number(s).

Sequence data were deposited in GenBank under the following accession numbers: KM502966 (16S rRNA sequence) and LWHC00000000 (PGAP file).
